# Quantitative relationships between *lacZ* mutant frequency and DNA adduct frequency in Muta™Mouse tissues and cultured cells exposed to 3-nitrobenzanthrone

**DOI:** 10.1093/mutage/gew067

**Published:** 2017-01-17

**Authors:** Paul A White, George R Douglas, David H Phillips, Volker M Arlt

**Affiliations:** 1 Environmental Health Science and Research Bureau, Health Canada, Tunney’s Pasture, Colombine Driveway, Ottawa, Ontario, Canada; 2 King’s College London, Analytical and Environmental Sciences Division, MRC-PHE Centre for Environment and Health, Franklin-Wilkins Building, London, UK

## Abstract

The frequency of stable DNA adducts in a target tissue can be used to assess biologically effective dose; however, the utility of the metric in a risk assessment context depends on the likelihood that the DNA damage will be manifested as mutation. Previously, we employed the Muta™Mouse system to examine the induction of *lacZ* mutants and DNA adducts following exposure to the well-studied mutagenic carcinogen 3-nitrobenzanthrone (3-NBA). In this follow-up work, we examined the empirical relationships between total adduct frequency and mutant frequency (MF) in tissues and cultured cells following acute 3-NBA exposure. The results show a significant induction of DNA damage and *lacZ* mutants in liver, colon and bone marrow, as well as FE1 pulmonary epithelial cells. In contrast, lung and small intestine samples had low, but significantly elevated adduct levels, with no significant increases in *lacZ* MF. Additional analyses showed a significant relationship between the *mutagenic efficiency of total adducts*, measured as the slope of the relationships between MF and total adduct frequency, and tissue-specific mitotic index (MI). The lack of mutation response in lung, in contrast to the high *in vitro* MF in FE-1 lung cells, is likely related to the 100-fold difference in MI. The lack of small intestine mutagenic response may be related to limited metabolic capacity, differences in DNA repair, and /or chemically induced apoptosis that has been observed for other potent mutagens. The results indicate that interpretation of adduct frequency values in a risk assessment context can be improved by considering the MI of the target tissue; however, more generalised interpretation is hampered by tissue-specific variations in metabolic capacity and damage processing. The work provides a proof of principle regarding the use of the Muta™Mouse system to critically examine the health risks associated with tissue-specific adduct loads.

## Introduction

Chemical modifications of nucleotides are a major cause of DNA damage and mutation; publications documenting the reaction of chemical mutagens with DNA date back to the late 1940s when Elmore *et al.* (1) showed that mustard gas, *bis*(2-chloroethyl)sulfide, reacts with DNA *in vitro*. Later work demonstrated that mustard gas induces *N*7-guanine alkylations in mouse DNA *in vivo* (2). In due course, analysis of covalently linked base modifications, commonly referred to as DNA adducts, became an essential tool for mechanistic investigations of chemical mutagens and environmental carcinogens.

In an *in vivo* context, the observed frequency of DNA adducts in a given tissue can be employed as a marker of internal dose and target tissue exposure (i.e., biologically effective dose). Adduct frequency at a given post-exposure time reflects the amount of a mutagen, or its reactive metabolite, that is delivered to target tissues, reacts with DNA to form chemically stable products, and has not been removed by DNA repair. Thus, adduct frequency can be employed as a useful biomarker along the continuum from exposure to pathophysiologic outcome (3,4). Moreover, it has become increasingly clear that the frequency of stable, promutagenic DNA adducts in a target tissue may also have a role in facilitating risk assessment of mutagenic carcinogens in environmental and occupational settings and, as such, DNA adduct analysis has become an essential tool in molecular epidemiology (5,6). However, since regulatory decisions are based on mutagenic activity and induced mutation frequency (MF), rather than DNA damage and adduct frequency, there is ongoing debate regarding the utility of adduct frequency metrics in a health and disease risk context. More specifically, since there are several steps between adduct formation and mutation, it is generally acknowledged that adduct frequency can only be used as a dosimeter of internal dose, and hence, a biomarker of potential effect (7,8). The context for the use of DNA adducts in assessing disease risk has been addressed in several excellent reviews (7,9–16).

Preston and Williams (10) and Jarabek *et al*. (9) describe key events in the DNA reactive mode-of-action (MOA) for suspected human carcinogens. The framework presented and discussed in these reviews recognises that the characteristics of a cancerous cell can be acquired via genetic mutation, and DNA damage can initiate the aforementioned multistep process resulting in mutation. Mutation fixation requires misreplication of the damaged template or misrepair of damaged DNA, and the multi-step cellular response to DNA damage, which involves complex dynamic interplay of damage, repair and replication, determines the likelihood and nature of mutations (17). The process is eloquently discussed in the aforementioned works by Jarabek *et al.* (9) and Preston and Williams (10); in addition, Holmquist and Gao (18) provide a quantitative framework describing the ‘sequential steps of mutagenesis’, a concept initially presented by Thilly (19). Holmquist and Gao formulate the probability of a chemically induced mutation at a particular position along a gene sequence as the product of several key metrics, including the probability that the base will be chemically altered (damaged), the probability that the damage will not be repaired, and the probability that the damaged site will be misread by a polymerase.

The probability that a DNA adduct will elicit a mutation is sometimes referred to as ‘mutagenic or mutational efficiency’ (9,20,21). According to Jarabek *et al.* (9), the mutagenic efficiency associated with a specific type of chemically induced DNA damage (i.e., adduct) is determined by a variety of biochemical, physiological and genetic factors, including the type, chirality, local sequence context and stability of the adduct, the cell-, tissue- or organ-specific repair capacity, the frequency of cellular replication, the background level of damage, and the likelihood of replicative by pass.

In contrast to DNA adducts, the health risks associated with the induction of mutations above background levels are well established, and mutagenesis has been empirically and mechanistically linked to cancer and heritable genetic diseases (22–24). Consequently, induction of mutation and/or chromosomal damage is routinely used as an endpoint for the regulatory evaluation of industrial chemicals, food additives and therapeutic products (25–27). However, the most common regulatory *in vivo* assays employed to assess mutagenic activity are restricted to haematopoietic cells (e.g., bone marrow, reticulocytes, mature red blood cells), and indicators of DNA damage (e.g., adducts, unscheduled DNA synthesis, etc.) are used to assess exposure of target and non-target tissues, and/or to provide information on the mode of action for suspected carcinogens. More recently, transgenic rodent assays (e.g., Muta™Mouse, Big Blue^®^ Mouse and Rat, lacZ Plasmid Mouse, gpt delta mouse and rat) have afforded the ability to assess mutation induction in virtually any tissue; and moreover, investigate empirical relationships between DNA damage frequency and induced MF in the same animals and tissues (28,29). This type of analysis can permit an evaluation regarding the utility of DNA adduct frequency; more specifically, it’s potential to predict the likelihood of mutations in various tissues following exposure to environmental mutagens and/or mutagenic carcinogens.

3-Nitrobenzanthrone (3-NBA) is a carcinogenic product of diesel combustion (30) that is highly mutagenic *in vitro* and *in vivo*. It induces squamous cell carcinomas in the lungs of F344 rats following intratracheal instillation (31), but did not initiate tumour formation in NMRI mouse skin (32). Mechanistic investigations have shown that the nitro group is reduced by cytosolic nitroreductases such as NAD(P)H:quinone oxidoreductase (33–35). The reduced metabolite, 3-aminobenzanthrone (3-ABA), is predominantly activated by cytochrome P450 (CYP) isozymes 1A1 and 1A2, and both 3-NBA and 3-ABA can yield an unstable hydroxylarylamine intermediate (i.e., *N*-hydroxy-3-aminobenzanthrone or *N*-OH-3-ABA) (36). *N*-acetyltransferases (NATs) and sulfotransferases (SULTs) can further metabolise the unstable intermediate to ultimately yield highly reactive cations (e.g., nitrenium or carbenium) (33,35). The reactive metabolites of 3-NBA and 3-ABA have been shown to form several stable DNA adducts (37–39). The average human intake of 3-NBA by inhalation is ~90 pg/day; however, intake by populations in areas of high air pollution can be considerably greater (33).

Three of the five spots detected in the autoradiographic profiles of ^32^P-postlabelled 3-NBA-induced DNA adducts have been identified as dA-*N*^6^-3-ABA (Spot 1), dG-*N*^2^-3-ABA (Spot 3) and dG-C8-*N*-3-ABA (Spots 4/5) (37,38), and Arlt *et al.* (36–38,40,41) have shown that 3-NBA-DNA adduct profiles are the same for a variety of murine tissues. Bieler *et al.* (42) and Kawanishi *et al.* (43) investigated the formation and persistence of 3-NBA adducts, and noted that dG-*N*^2^-3-ABA adducts are the most persistent. Results showed that 85% of dG-*N*^2^-3-ABA adducts remain after 24 hrs, and they are still detectable 20 weeks after treatment (Supplementary Table 1, available at *Mutagenesis* Online). This suggests that they are less likely to be repaired (43), perhaps by avoidance of global genomic nucleotide excision repair (44), and consequently more likely to contribute to induction of mutations. Arlt *et al.* (37) note that the persistence and relative prevalence of this adduct, as well as dG-C8-*N*-3-ABA, is consistent with the earlier observation (45) of the induction of GC to TA transversions in the *cII* gene of Muta™Mouse liver following *i.p.* exposure. This is consistent with the works of Kawanishi *et al.* (43,46) and Pande *et al.* (17) that employed site specific mutagenicity assays to show that the dG-*N*^2^-3-ABA (Spot 3) and dG-C8-*N*-3-ABA (Spots 4/5) adducts are promutagenic, inducing GC to TA transversions in both bacterial and human cells. Previously, we documented the formation of stable DNA adducts and *lacZ* transgene mutations in Muta™Mouse *in vivo* and Muta™Mouse cells *in vitro* following different exposures to 3-NBA (37). In this communication we examine the empirical relationships between total adduct frequency and total MF in Muta™Mouse tissues (i.e., liver, lung, colon, small intestine and bone marrow) and cultured Muta™Mouse cells exposed to 3-NBA.

## Materials and methods

### Test compound

3-Nitro-7H-benz[*de*]anthracen-7-one (3-nitrobenzanthrone or 3-NBA) (CAS No. 17117-34-9; purity 99%) was obtained from the Sigma Library of Rare Chemicals (Sigma-Aldrich, Oakville, Ontario, Canada).

### Animal treatment and tissue collection

Transgenic animals (strain 40.6; BALB/c × DBA/2) were obtained from a breeding colony maintained at the Health Canada Animal Care Facility. Animals were maintained and treated under conditions approved by the Health Canada Animal Care Committee. Male mice (16–20 weeks) were maintained on a 12-h light–dark cycle and provided with fresh water and Rodent Chow (Ralston Purina, Hazleton, PA, USA) *ad libitum*. Each experiment involved up to five animals per dose group, and the data presented were compiled from two separate rounds of exposure. The maximum number of replicate animals per dose group was 8, the minimum was 5. A single dose of 25, 50 or 75 mg/kg body weight 3-NBA in olive oil was administered by oral gavage. Following the single treatment, tissues were collected at 18 h for analysis of DNA adducts in all tissues and *lacZ* transgene mutations in bone marrow and intestinal epithelium, at 3 days for analysis of *lacZ* mutations in bone marrow, intestinal and colonic epithelium and liver, and at 28 days for analysis of *lacZ* mutations in liver, intestinal and colonic epithelium, and lung. Mice were killed by cervical dislocation and liver, lung, colon, small intestine and bone marrow were removed, frozen in liquid nitrogen and stored at ‒80°C until DNA isolation. The caecum was routinely used to locate the end of the intestine and the start of the colon. To obtain bone marrow, femurs were flushed with cold phosphate-buffered saline (PBS), the solution centrifuged at 10000g for 1 min and the pellet stored at ‒80°C. Colonic and intestinal epithelium were rinsed with cold PBS to remove luminal contents, and the epithelial layer stripped from supporting tissue by inverting the tissue in 0.075 M KCl with 20 mM EDTA and repeatedly forcing it in and out of a needleless 5-ml syringe (47). This processing permitted liberation of the epithelial layers, and significantly reduced the likelihood of microbial contamination of murine DNA preparations. Minced or homogenised tissues were digested overnight in lysis buffer, and the DNA extracted and handled as previously described (48,49). Table 1 provides a summary of the tissue analyses conducted.

**Table 1.** Summary of Muta™Mouse tissue analyses following a single gavage administration of 3-NBA

Tissue	Post-exposure sampling time		
	18 h	3 days	28 days
Bone marrow	Adducts, *lacZ* mutations	*lacZ* mutations	
Intestinal epithelium	Adducts, *lacZ* mutations	*lacZ* mutations	*lacZ* mutations
Colonic epithelium	Adducts	*lacZ* mutations	*lacZ* mutations
Liver	Adducts	*lacZ* mutations	*lacZ* mutations
Lung	Adducts		*lacZ* mutations

### FE1 cell culture and treatment

FE1 is a stable epithelial cell line derived from Muta™Mouse lung (49). Cells were cultured in a 1:1 mixture of Dulbecco’s modified Eagle’s medium and F12 nutrient mixture supplemented with 2% (v/v) foetal bovine serum, 100 U/ml penicillin G, 100 mg/ml streptomycin sulphate and 1 ng/ml murine epidermal growth factor (EGF) (GIBCO–Invitrogen, Burlington, Ontario, Canada).

2–3 × 10^5^ cells were seeded on 100-mm culture dishes and incubated overnight to ~20% confluence. The following morning cells were exposed for 6 h to a series of 3-NBA concentrations in serum-free medium, or a solvent control in serum-free medium. Cells were then washed twice with PBS and incubated for 72 h (i.e., 72 h sampling time). Genomic DNA was isolated as described previously (50,51). Briefly, cells were incubated overnight at 37°C in lysis buffer containing 1% sodium dodecyl sulphate and 1 mg/ml fresh proteinase K (GIBCO–Invitrogen). Lysates were extracted with phenol–chloroform (1:1) followed by chloroform, and sodium chloride added to a final concentration of 0.2 M. The DNA was precipitated in two volumes of ethanol, spooled onto a sealed Pasteur pipette, washed in 70% ethanol and dissolved in 15–100 µl of TE buffer (10 mM Tris and 0.1 mM EDTA, pH 7.6).

### *LacZ* mutation analysis

Total transgene mutant frequency (MF) was determined using the P-gal-positive selection assay described elsewhere (28,51). Briefly, λgt10*lacZ* copies were rescued from genomic Muta™Mouse DNA using the Transpack™ system (Agilent Technologies, Mississauga, Ontario, Canada). Packaged phage preparations were mixed with host bacteria (50) and allowed to adsorb for 25 min at room temperature. An aliquot of the phage-bacteria mix was diluted and plated on non-selective minimal agar to determine total titre (i.e., plaque forming units or pfu). The remaining phage-bacteria mixture was plated on minimal agar with the selective agent, 0.3% w/v phenyl-ß-D-galacto-pyranoside (P-gal; Sigma-Aldrich). Both were incubated overnight at 37°C. Total MF was expressed as the ratio of mutant plaques to total pfu. Mutant plaques were not collected for *lacZ* sequencing; hence, MF values are not clonally corrected.

### DNA adduct analysis using ^32^P-postlabelling

DNA adducts were measured using the butanol enrichment version of the ^32^P-post-labelling method as described previously (52,53), with minor modifications. DNA samples from the mutation analyses (4 µg) were digested with micrococcal nuclease (120 mU, Sigma-Aldrich, Gillingham, UK) and calf spleen phosphodiesterase (40 mU, Calbiochem, Nottingham, UK), extracted with butanol and labelled as reported. Chromatographic conditions for thin-layer chromatography (TLC) on polyethyleneimine-cellulose (Macherey-Nagel, Düren, Germany) were D1, 1.0 M sodium phosphate, pH 6.0; D2, 4 M lithium formate and 7 M urea, pH 3.5 and D3, 0.8 M lithium chloride, 0.5 M Tris and 8.5 M urea, pH 8.0. DNA adduct levels (relative adduct labelling or RAL) were calculated from the adduct counts per minute (cpm), the specific activity of [γ-^32^P]ATP and the amount of DNA used. Results were expressed as DNA adducts per 10^8^ nucleotides (nt). In all cases, RAL values represent the sum of a cluster of 5 adduct spots detected via TLC ^32^P-postlabelling (see (37,38,40)). In some cases limitations in the amount of DNA obtained from certain tissues (e.g., bone marrow), and the low levels of 3-NBA-induced adducts, restricted the ability to detect adducts in all replicates. Consequently, although most dose-tissue combinations yielded 5 replicate measurements, some bone marrow samples (e.g., 25 mg/kg) yielded only 2 replicate measurements.

### Data analysis

All raw data are available from the corresponding author on request.

Not all endpoints were assesses for all samples (see Table 1). For most dose-tissue combinations 5–8 biological replicates were analysed for *lacZ* MF, and five replicates analysed for RAL. In some cases, DNA adduct levels were near the detection limit and fewer than five replicates yielded reliable values (e.g., bone marrow). *In vitro lacZ* MF results are based on 2 (0.1 µg/ml only) to 8 replicates; only two replicates per dose were analysed for DNA adduct levels.

MF and RAL data were analysed via Poisson regression using SAS version 9.1 (SAS Institute, Cary, NC, USA). For MF analyses the natural log of total plaque count was used as an ‘offset’ (i.e., regression variable with a constant coefficient of 1.0 for each observation). Log-linear relationships between mutant count or RAL and concentration or dose were specified by a natural log link function. Type 1, or sequential analysis, was employed to examine the statistical significance of the chemical treatment, and post hoc custom contrasts statements were employed to evaluate the statistical significance of responses at selected doses or concentrations. Ordinary least-squares linear regression analysis was employed to investigate empirical relationships between MF for a selected tissue and sampling time and total RAL in the same tissue at 18 h sampling time. Since the numbers of observations for each endpoint in a given tissue (i.e., total RAL and MF) were not identical and varied between 2 and 8, unbiased analyses of the dose-matched empirical relationships between total RAL and MF required repeated random subsampling by dose, and subsequent iterative regression analysis (i.e., bootstrapping or Monte Carlo analysis). One thousand sampling iterations, with subsequent regressions, provided unbiased statistical metrics, including the bootstrapped slope of the relationship between MF and total RAL (i.e., *the mutagenic efficiency of total adducts*) as well the bootstrapped *r*^2^, F ratio and ANOVA *P* value. Subsequent analysis of variance was employed to compare mean slopes across tissues, and ordinary least-squares linear regression analysis was employed to investigate the empirical relationship between the mutagenic efficiency of total adducts and mitotic index (i.e., the ratio of the number of cells in mitosis to the total number of cells in a population). Since the Muta™Mouse MF analysis did not investigate the mutagenicity of individual adducts, it was not possible to investigate adduct-specific mutagenic efficiency (9,20,21). Since tissue-specific MI (mitotic index) values are not available for the Muta™Mouse, adult rodent values were collected from the scientific literature. Mean values used for the presented analyses are based on data collected from a minimum of four separate studies per tissue (54–67). Since the number of MI values varied from a low of 4 (lung) to a high of 15 (liver), it was necessary to employ the aforementioned Monte Carlo approach to obtain an unbiased assessment of the relationship between the mutagenic efficiency of total adducts and MI. Regression diagnostics (e.g., Studenised residuals, leverage values) were employed to examine the influence of individual observations on the outcome of the analyses (68). Individual predictions of mutagenic efficiency for a given MI employed the method described by Gujarati (69).

## Results

Previous work demonstrated a disconnect between the levels of total 3-NBA-induced DNA adducts and the levels of induced transgene mutations in several tissues, for a variety of short- and longer-term exposure regimens. The present study investigated the empirical relationship between total DNA adduct levels in selected tissues observed 18 h following a single oral administration of 25, 50 or 75 mg/kg 3-NBA, and total MF in the same tissues 18 h (bone marrow, small intestine), 3 days (bone marrow, liver, small intestine, colon) or 28 days (bone marrow, liver, small intestine, colon, lung) after treatment. The *in vivo* RAL results observed at 18 h after a single acute dose are new data, while the MF data include new data, as well as those previously published (37,48). In addition, investigation of the MF-RAL relationship in the present study incorporates published data on RAL and MF in FE1 cells following *in vitro* exposure to 0.1, 1, 3 and 10 µg/ml (37).

**Figure 1.**
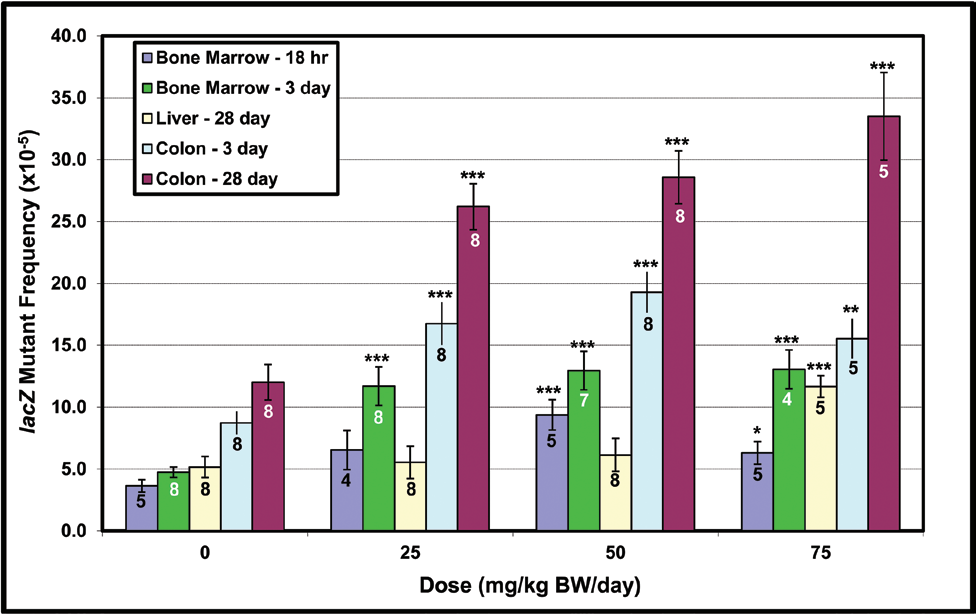
Mean *lacZ* mutant frequency (MF) in selected Muta™Mouse tissues following a single oral 3-NBA administration of 25, 50 and 75 mg/kg. Sampling times ranged from 18 h (bone marrow) to 28 days (liver and colon). For all results shown Poisson regression revealed a significant treatment effect with *P* < 0.001 (Type 1 chi-square). Asterisks show the results of custom contrasts between individual doses and the concurrent control (based on the asymptotic chi-square distribution of the likelihood ratio). *P < 0.05, **P < 0.01, ***P < 0.001. Error bars show one standard error of the mean. Values on each bar show the number of biological replicates (animals). No significant increase in MF observed for the following, which are not shown: colonic epithelium (18 h), liver (3 days), lung (28 days) and intestinal epithelium (18 h, 3 and 28 days).

The *in vivo lacZ* transgene mutagenicity results (Figure 1) show statistically significant induction of mutations in bone marrow (18-h and 3-day sampling), liver (28-day sampling), and colonic epithelium (3- and 28-day sampling). The most marked increases were observed in colon, followed by bone marrow and liver. No significant increases in MF were observed in colonic epithelium (18-h sampling), liver (3-day sampling), lung (28-day sampling) and intestinal epithelium (18-h, 3- and 28-day sampling). With respect to *in vitro lacZ* transgene mutagenicity, the results presented in Arlt *et al.* (37) showed a significant increase in MF at 1, 3, and 10 µg/ml. The 0.1 µg/ml concentration did not elicit a significant increase in MF.

**Figure 2.**
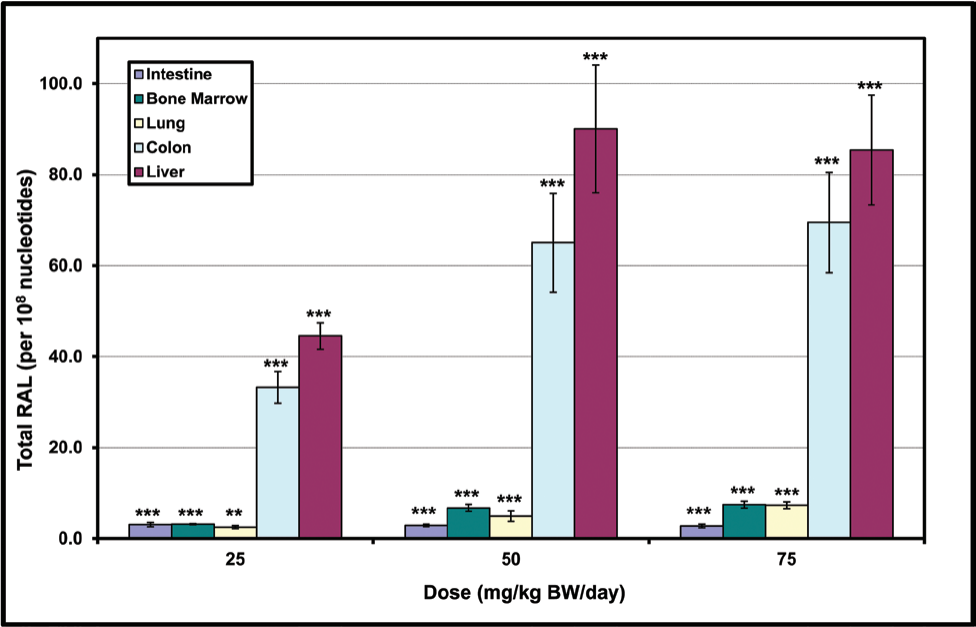
Mean total RAL (relative adduct labelling) in selected Muta™Mouse tissues following a single oral 3-NBA administration of 25, 50 and 75 mg/kg. All observations were made 18 h after dosing. Adducts were not detected in any of the control animals. For all results Poisson regression showed a significant treatment effect with *P* < 0.001 (Type 1 chi-square). Asterisks show the results of custom contrasts between individual doses and the concurrent control (based on the asymptotic chi-square distribution of the likelihood ratio). **P < 0.001, ***P < 0.0001. Error bars show one standard error of the mean. All means are based on five biological replicates, except bone marrow 25 mg/kg for which *n* = 2.

**Figure 3.**
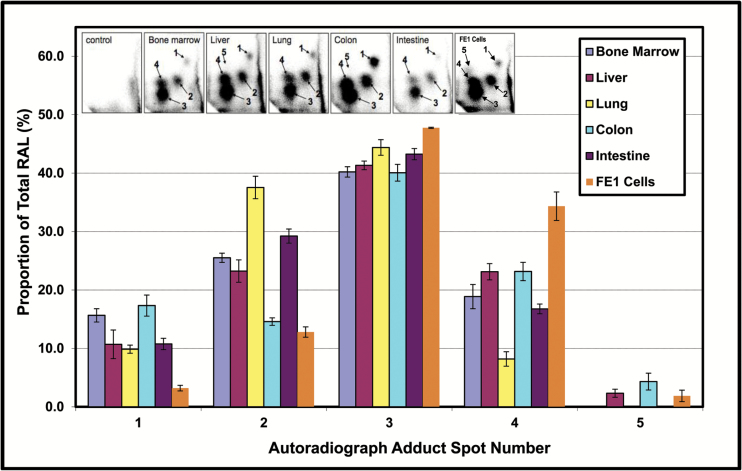
The relative abundance of each 3-NBA DNA adduct in the autoradiographic profile (i.e., spots 1–5). Adduct profiles for indicated *in vivo* tissues represent the single oral dose of 50 mg/kg 3-NBA. The adduct profile for FE1 cells represents the 3 µg/ml 3-NBA treatment. RAL, relative adduct labelling. For *in vivo* tissues, *N* = 5. For *in vitro* FE1 cells, *N* = 2. Error bars show one standard error of the mean. The inset shows typical autoradiograms illustrating the positions of spots 1 through 5 (i.e., 50 mg/kg 3-NBA *in vivo* and 3 µg/ml 3-NBA *in vitro*). The identities of the individual adducts are described in the text. Detailed summaries of adduct proportions for all samples across all doses are provided in Supplementary Figure 1, available at *Mutagenesis* Online.

The *in vivo* RAL results show a statistically significant increase in adduct frequency 18 h after a single oral dose for bone marrow, liver, intestinal and colonic epithelium and lung (Figure 2). The greatest increases in total RAL were seen in liver and colon, with an apparent plateau at 50–75 mg/kg. Autoradiographic profiles showed a pattern of five adduct spots that has been reported previously (37). No adducts were detected in control tissues (see Figure 3). Earlier studies characterised the structure of these adducts, which have been identified by HPLC analyses of individual spots and mass spectrometry using authentic standards (38,39). Arlt *et al*. (38), which employed the same ^32^P-postlabeling methodology as that employed herein, prepared 3-NBA-derived DNA adducts and used them as authentic standards to determine the identity of adducts formed *in vivo*. More specifically, three of the spots were identified as 2-(2′-deoxyadenosin-*N*^6^-yl)-3-aminobenzanthrone (dA-*N*^6^-3-ABA, spot 1), 2-(2′-deoxyguanosin-*N*^2^-yl)-3-aminobenzanthrone (dG-*N*^2^-3-ABA, spot 3), and *N*-(2′-deoxyguanosin-8-yl)-3-aminobenzanthrone (dG-C8-*N*-3-ABA, spots 4/5) (38). The adduct profiles indicate that the dG-*N*^2^-3-ABA adduct (spot 3) is always the most common, accounting for between 37% (bone marrow, 25 mg/kg) and 46% (lung, 75 mg/kg) of the total RAL. Spot 2, an as-yet-unidentified dA adduct, and dG-C8-*N*-3-ABA (spots 4/5) are the next most common adducts, accounting for approximately 15% (colon, 25 and 50 mg/kg) to 38% (lung, 25 and 50 mg/kg), and 8% (lung, 25 and 50 mg/kg) to 23% (colon, 25 and 50 mg/kg, liver, 50 mg/kg), respectively. A typical distribution of adduct proportions in each tissue, as observed at 50 mg/kg, is shown in Figure 3. A detailed overview of adduct proportions for all tissues and all doses is presented in Supplementary Figure 1, available at *Mutagenesis* Online.

With respect to the previously published *in vitro* RAL results (37), a significant increase in RAL was observed at 0.1, 1, 3, and 10 µg/ml (i.e., averages of 5.3, 10.2, 128.6, 173.1 total RAL per 10^8^ nucleotides, respectively). No adducts were detected in the unexposed cells, and the adduct profile is similar to that observed *in vivo* (Figure 3a and b in [37]). Interestingly, the maximum adduct levels observed in FE1 cells are greater than those observed in some Muta™Mouse tissues (e.g., lung, intestine, bone marrow). More specifically, the maximum adduct levels observed in intestine, bone marrow and lung (i.e., 7.5, 3.1 and 6.4, respectively) are in the same range as that observed for *in vitro* exposures to less 1 µg/ml. In contrast, the maximum adduct levels observed in liver and colon (i.e., 88.0 and 72.2, respectively) are comparable to levels observed for *in vitro* exposures of 3 µg/ml. Figure 3 also illustrates the typical distribution of adduct proportions *in vitro* observed at 3 µg/ml. Similar to the *in vivo* results, the results show that the dG-*N*^2^-3-ABA adduct (spot 3) is predominant, followed by spots 4/5 and 2, decreasing in that order. Interestingly, the *in vitro* FE1 frequencies of spot 1 (i.e., lower) and 4 (i.e., greater) differ from what was observed *in vivo*. A detailed overview of adduct proportions for FE1 cells at all concentrations is provided in Supplementary Figure 1, available at *Mutagenesis* Online.

In summary, Figure 3 shows the predominance of the dG-*N*^2^-3-ABA adduct (spot 3) in all tissues as well as FE1 cells; spots 2 and then 4/5 (dG-C8-*N*-3-ABA) are generally the next most prevalent, respectively. Exceptions to this overall pattern are spot 1 in FE1 cells (low), and spot 4 in FE1 cells (high) and lung (low). The frequency of spot 2 is quite variable ranging from a low of 12.8 and 14.6% for FE1 cells and colon, respectively, to a high of 38% in lung.

Interestingly, the dose-related increases in total MF and total adducts (Figures 1 and 2) indicate that some of the dose–response relationships are distinctly non-linear, reaching zero order kinetics at elevated 3-NBA doses (e.g., bone marrow MF, liver RAL, etc.). Detailed investigations regarding the nature of 3-NBA dose–response relationships are outside the scope of this work, which addresses the relationships between total MF and total RAL across a series of matched doses. Numerous publications discuss the functional relationships between induced mutations and/or adducts and 3-NBA dose (39,44,70,71).

**Figure 4.**
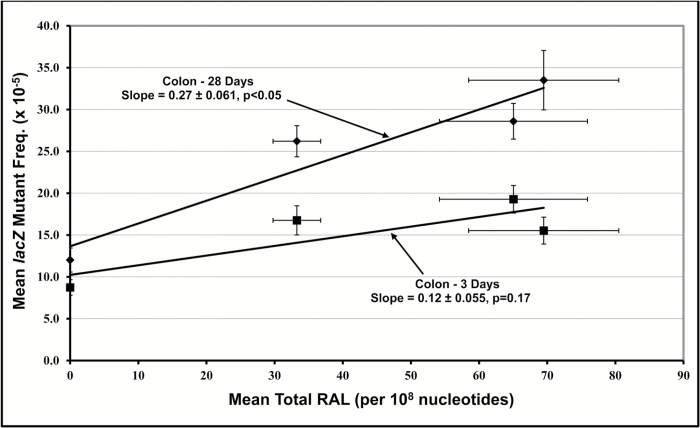
The empirical relationship between mutant frequency (MF) and total RAL (relative adduct labelling) in colonic epithelium. All RAL observations were made 18 h after treatment. MF observations were made 3 and 28 days after treatment. No significant increase in MF was observed 18 h after treatment. Error bars show one standard error of the mean. Slope values were calculated using ordinary least-squares linear regression.

**Figure 5.**
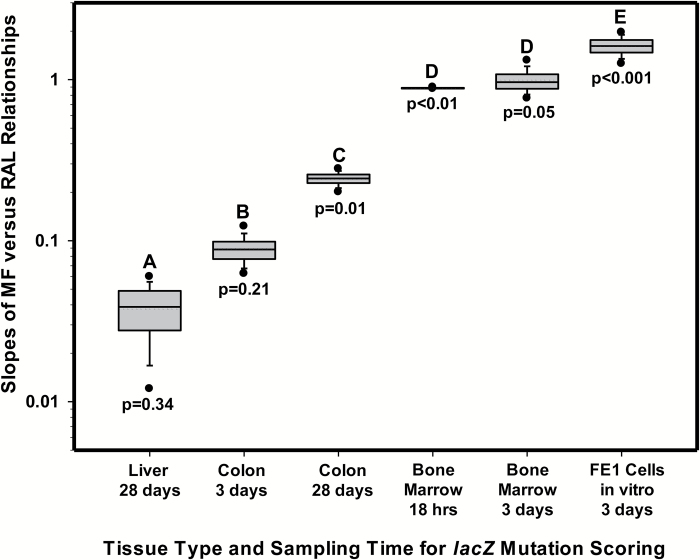
Distribution of bootstrapped slope values for the relationships between mutant frequency (MF) and total RAL (relative adduct labelling) for each tissue and sampling time combination that yielded a significant response. Slope values were calculated via least-squares regression on data selected using a sub-sampling routine that randomly chose MF and RAL values at each dose. Each distribution represents 1000 iterations of the sampling routine and slope calculation. The box limits represent the interquartile range (i.e., 25th and 75th percentiles), and the whiskers show the 5th and 95th percentiles. Symbols located beyond the whiskers are outliers. The solid line is the median value and the dotted line the arithmetic mean. All mean slope values (Table 2) are significantly greater than zero. *P* values show the ANOVA probability for rejection of the null hypothesis that total MF is not empirically related to total RAL. Letters above plots show the results of post hoc contrasts. When boxes are accompanied by the same letter, the mean slope values (i.e., mutagenic efficiency of total adducts) are not significantly different at *P* < 0.05.

Least-squares linear regression was employed to examine empirical relationships between MF and total RAL in tissues/cells that yielded significant dose-related increases. The results for colonic epithelium are illustrated in Figure 4. The difference in the slopes of the relationship between mean MF and mean total RAL indicate a higher total *yield* of *lacZ* mutants for 28-day sampling in comparison with 3-day sampling. Although these results are interesting, analysis of the relationship between average MF and average total RAL does not maximise the number of degrees of freedom for linear regression analysis and slope determination. However, variation in the number of total RAL and MF observations for each dose complicates use of the entire dataset for each tissue-sampling time combination. Accordingly, a repeated random sub-sampling routine (i.e., bootstrapping) was employed to maximise the use of the data and provide unbiased estimates of the slope of the MF-RAL relationships. For a given tissue and sampling time, the bootstrapping routine randomly selected appropriately sized groups of replicates for MF and total RAL at each dose, and then employed least-squares regression to determine the slope. One thousand iterations of this process provided a distribution of slope values. The results obtained, which are displayed in Figure 5, show that the average bootstrapped slope of the MF-RAL relationship, a metric henceforth termed *the mutagenic efficiency of total adducts,* is lowest in liver (28 days), greater in colon (3 and 28 days) and bone marrow (18 h and 3 days), and finally highest in FE1 cells exposed *in vitro*. Importantly, since the Muta™Mouse assay only permits the detection of total MF, it was not possible to examine the *mutagenic efficiency* of individual adducts (9,20,21). Table 2 summarises the results of the bootstrapped linear regression results for each tissue and sampling time combination that yielded significant increases in both MF and total RAL. Although all slope values are significantly greater than zero, indicating that total MF increases with increasing total RAL, the bootstrapped F ratios indicate that the ANOVA results for colon (3-day sampling) and liver are not significant at p<0.05. This incongruence suggests that, for some tissues, additional statistical power (e.g., additional doses and/or replicates) is required to conclusively demonstrate relationships between total MF and total RAL (i.e., effectively reduce the likelihood of a Type II error).

**Table 2.** Summary of bootstrapped linear regression results for the relationships between *lacZ* MF and total RAL

Tissue/Sampling Time	Mean Slope ± SEM	Mean r^2^	Mean F Ratio	df	ANOVA Mean *P* value
Liver: 28-day sampling	0.038 ± 0.014*	0.19	0.64	18	0.34
Colon: 3-day sampling	0.089 ± 0.018***	0.29	1.59	18	0.21
Colon: 28-day sampling	0.24 ± 0.023***	0.66	7.74	18	0.011
Bone marrow: 18-h sampling	0.89 ± 0.0059***	0.76	13.25	10	0.0043
Bone marrow: 3-day sampling	0.99 ± 0.17***	0.51	4.09	15	0.050
FE1 cells: 3-day sampling	1.62 ± 0.21***	0.83	22.34	9	0.00087

The results shown, which represent the average of 1000 regression iterations, are provided for each tissue and sampling time. df indicates the degrees of freedom associated with each regression.

SEM, standard error of the mean.

*P < 0.05, **P < 0.01, ***P < 0.001.

**Figure 6.**
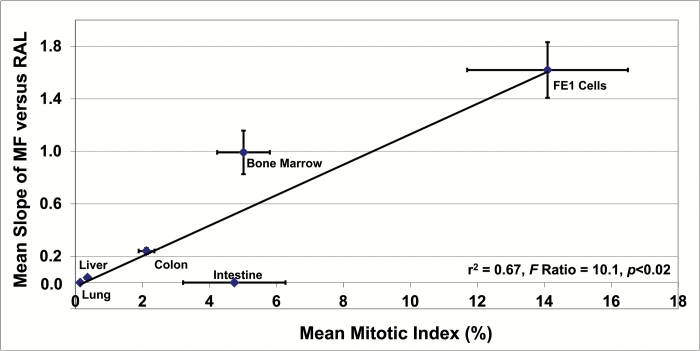
The effect of mitotic index on the slope of the relationships between mutant frequency (MF) and RAL (relative adduct labelling) for different tissues/cells. No *lacZ* mutants were detected in lung and small intestine, and the MF-RAL slopes for these tissues are zero. The inset shows the results of bootstrapped linear regression analysis. Exclusion of the small intestine observation yields slightly different results (see text). Vertical and horizontal error bars show one standard error of the mean. Where error bars cannot be seen, the bars were smaller than the plotting symbol. Mitotic index values, which were collected from the published literature, are expressed as the percentage of examined cells undergoing DNA replication and/or mitosis. Four to fifteen values were collected for each tissue shown; values represent several strains of rodents and multiple adult ages. The majority of studies employed microscopic analyses of Geimsa or H&E (hematoxylin and eosin) stained tissues. A small number of studies used autoradiography to quantify the proportion of cells in S-phase.

In their review that discusses the use of DNA adduct data in cancer risk assessment, Jarabek *et al.* (9) note that the likelihood that a particular DNA adduct or adduct load will result in mutation, a concept referred to as mutagenic efficiency, is dependent on a number of factors including the frequency of cellular replication. The mitotic index (MI) of cells in several tissues has been determined in numerous rodent studies, and a review of published values (*N* = 4–15 per tissue) provided averages of 0.36 ± 0.07%, 2.1 ± 0.2%, 0.14 ± 0.04, 4.8 ± 1.5, and 5.0 ± 0.8% for liver, colonic epithelium, lung, small intestine and bone marrow, respectively (54–67). Although tissue-specific MI values for the Muta™Mouse are not available, the MI for the Muta™Mouse FE1 cell line is 14.1 ± 2.4% (49). Although the above *in vivo* values do not account for variations among cell populations in a given tissue (e.g., crypt position, different haematopoietic cells, etc.), they correlate with the maximum slope of the observed empirical relationships between MF and total RAL for liver, colon, bone marrow and FE1 cells. The results, which are illustrated in Figure 6, show a significant positive effect of mitotic index on the maximum observed mutagenic efficiency of total adducts. Although a significant increase in *lacZ* MF was not observed for lung, and concomitantly, the slope of MF-total RAL relationship for lung is zero, the low MI of lung cells (72) suggests that the slope of the MF-total RAL relationship would be lower than liver and extremely shallow. The lack of increase in MF in small intestine also results in a zero slope for the MF-total RAL relationship. The bootstrapped linear regression analysis based on all observations (Figure 6) revealed a significant positive relationship (i.e., *r*^2^ = 0.67, *F* Ratio = 10.1, *P* < 0.02). As expected, the intercept is not statistically different from zero (i.e., 0.05 ± 0.12). Examination of regression diagnostics (e.g., leverage and Studentised residuals) was employed to assess model misspecification and the influence of individual observations. Residual value analyses did not support any notion of model misspecification, and leverage (i.e., *h*_*i*_) analyses revealed that the FE1 observation is influential. This is not at all surprising given the distance of its MI from the mean MI across all tissues/cells. Importantly, the Studentised residual for the FE1 observation is exceptionally small, revealing that there is no objective justification for its removal from the dataset. On the contrary, the value is in line with observations for lung, liver and colon (i.e., increase in the mutagenic efficiency of total adducts is proportional to the increase in MI). Analysis of Studentised residuals and Studentised deleted residuals revealed that the small intestine observation is a marginally significant negative outlier (i.e., Studentised deleted residual = -2.46, *P* = 0.057). This justifies its removal for reanalysis of the data. Exclusion of small intestine from the regression analysis yields slightly different results (i.e., *r*^2^ = 0.85, *F* Ratio = 59.4, *P* < 0.002); however, the slopes of the two models are not significantly different (i.e., 0.098 ± 0.034 versus 0.110 ± 0.016).

## Discussion

Our earlier work showed that single oral doses of 25 and 50 mg/kg 3-NBA induced significant increases in *lacZ* MF in Muta™Mouse colonic epithelium, and in both bone marrow and colonic epithelium, respectively (48). However, single acute exposures did not elicit significant increases in *lacZ* MF in bladder or liver. The same study showed that 28-day repeat-dose oral exposures to 2 and 5 mg/kg induced significant increases in MF in bone marrow, and both bone marrow and liver, respectively. Also, repeated dose administration did not yield significant increases in MF in lung or intestinal epithelium. That study (48) employed a recommended sampling time of 3 days for bone marrow, 28 days for liver, lung, colonic and intestinal epithelium (73,74). Similarly, in another study, we also showed that a 28-day repeated dose oral exposure of 2 or 5 mg/kg, followed by a 28-day sampling time, induced a significant increase in MF in bone marrow and liver (37), but showed no significant increase in MF in lung and intestinal epithelium at any dose. Both studies (i.e., (37,48)) also showed a significant increase in MF in Muta™Mouse FE1 cells exposed to 3-NBA *in vitro* (0.1, 1, 3 and 10 µg/ml).

The 28-day repeated dose study showed significant levels of DNA adducts in liver, lung and bone marrow in Muta™Mouse (37). Moreover, significant levels of DNA adducts were detected in FE1 cells exposed to 3-NBA (37). These results, in conjunction with earlier rodent studies in Muta™Mouse, Wistar and SD rat, confirm significant increases in 3-NBA adducts in numerous tissues such as liver, lung, bone marrow, colon, intestine, bladder, stomach and kidney, but significant increases in MF only for selected tissues such as colon, liver and bone marrow (41,45,75).

Lambert *et al.* (28,29) noted that *in vivo* mutation detection models such as the Muta^™^Mouse can be used to examine virtually any tissue, with the response to a given dose in a given tissue being influenced by route of administration, toxicokinetics, tissue-specific DNA repair, tissue specific mitotic index and the nature of the genetic target. The Muta™Mouse system is well-suited to the analysis carried out in this study, since the multi-copy transgene (i.e., 29 ± 4 copies) (76) is neutral (i.e. transcriptionally inactive), and therefore, not subject to transcription coupled repair. Accordingly, by default, the dominant mode of DNA repair operating on 3-NBA adducts will be global genomic repair, a system that should be fully active during the course of the experiment. Interestingly, recent work by Kucab *et al.* (44) indicates that 3-NBA adducts may evade removal by global genomic nucleotide excision repair, and the authors suggest that resultant adduct persistence may be playing a significant role in 3-NBA mutagenicity.

The results obtained herein showed that a significant increase in total 3-NBA-DNA adducts can be detected in bone marrow, liver, intestinal and colonic epithelium and lung 18 h after oral administration of a single dose. Moreover, the most frequent adduct detected in all tissues/cells examined is dG-*N*^2^-3-ABA (Figure 3, Supplementary Figure 1, available at *Mutagenesis* Online, spot 3), a promutagenic adduct (17,43,46) that is approximately 4- to 9-fold more persistent than the other 3-NBA-induced adducts ((17,43); Supplementary Table 1, available at *Mutagenesis* Online). However, mutational analyses revealed significant increases in *lacZ* MF only for bone marrow, liver and colonic epithelium; significant increases in MF for lung and intestinal epithelium were not observed, despite significant elevation in adducts. The lack of *lacZ* mutants in the latter tissues included results from multiple (intestine) and/or extended (lung) sampling times ranging from 18 h to 28 days. Additionally, our earlier work showed that 3-ABA, the reduced metabolite of 3-NBA, also failed to induce a significant increase in *lacZ* mutations in Muta^™^Mouse lung (37). Interestingly, other researchers have detected significant increases in *lacZ* MF in lung and intestine following exposures to other homo- or heterocyclic aromatic compounds. For example, numerous studies have shown significant increases in lung MF, in some cases as high as 28-fold above concurrent control, following oral exposures to PAHs such as benzo[*a*]pyrene and dibenz[*a,h*]anthracene (Supplementary Table 2, available at *Mutagenesis* Online). Similarly, several studies have shown significant increases in small intestine MF, in some cases as high as 170-fold above concurrent control, following exposures to a range of homo- or heterocyclic aromatic compounds (Supplementary Table 2, available at *Mutagenesis* Online). Interestingly, the lung data summarised in Supplementary Table 2, available at *Mutagenesis* Online, includes data from a study by Kohara *et al.* (77) that documented a significant 1.6-fold increase in MF following 28-day repeat dose intragastric administration of a dinitropyrene mixture. Thus, the lack of 3-NBA-induced *lacZ* mutants in lung and small intestine observed in the current study can reasonably be regarded as atypical for the Muta^™^Mouse.

The results shown herein confirm that all examined tissues were exposed to the genotoxic metabolite(s) of 3-NBA (Figure 2). Moreover, despite some aforementioned exceptions (e.g., lung spots 2 and 4), there is minimal cross-tissue variation in the 3-NBA adduct profiles, and the predominant adduct in all samples examined is dG-*N*^2^-3-ABA (37). Nevertheless, although some tissues showed high levels of total adducts (e.g., liver, Figure 2), and a high proportion of the total comprised of an adduct that is known to be both persistent and promutagenic (Supplementary Table 1, available at *Mutagenesis* Online, (42,43,46)), the yield of *lacZ* mutants for some tissues was modest (e.g., liver, Figure 1) and clearly dependent on factors other than DNA damage frequency. Consequently, the mean slopes of the relationships between *lacZ* MF and total RAL varies considerably across tissues (Figure 5). For example, the results illustrated in Figure 5 and Table 2 indicate a 2.3-fold difference in slope between 28-day liver and 3-day colon, and a ~6-fold difference between 28-day liver and 28-day colon. This variation in the slope, also referred to as *the mutagenic efficiency of total adducts*, is likely dependent on tissue-specific factors, such as differential repair and/or differences in replicative capacity, which in turn influence mutation manifestation time (Figure 4). This notion is supported by the Lambert *et al.* summary of transgenic rodent mutagenicity data (28,29), which showed that extended sampling times for slowly dividing tissues (e.g., liver) generally yield higher induced mutant frequencies. Since maximum MF is a function of sampling time, it seems reasonable to hypothesise that tissue-specific cell turnover has a major influence on the yield of *lacZ* mutants in the samples analysed herein, and hence the mutagenic efficiency of total 3-NBA adducts. Indeed, the empirical relationship between the maximum observed mutagenic efficiency of total adducts and MI supports this hypothesis (Figure 6), which is consistent with earlier reports in the literature. In their analyses of 1,2,3-trichloropropane, La *et al.* (78) noted the importance of cell proliferation in understanding of the biological significance of DNA adduct frequency measurements. Ochiai *et al.* (79) showed that 2-amino-3-methylimidazo[4,5-*f*]quinoline (IQ)-DNA adduct levels do not correlate with *lacI* MF in Big Blue^®^ mouse tissues, and the authors asserted that observed MF is likely determined by both tissue-specific adduct levels and cellular proliferation rates.

The contention that tissue-specific cell turnover is having a strong influence on the yield of 3-NBA-induced *lacZ* mutants, and hence the mutagenic efficiency of total 3-NBA adducts, is consistent with the similarity in adduct profiles across the tissues examined. Little variations in adduct profiles across the tissues examined (Figure 3), and the predominance of the most persistence and promutagenic adduct (i.e., dG-*N*^2^-3-ABA, Spot 3) (Supplementary Table 1, available at *Mutagenesis* Online, (42,43,46)), also supports the notion that other factors, such tissue-specific cell turnover, are influencing the yield of 3-NBA-induced *lacZ* mutants, and hence the mutagenic efficiency of total 3-NBA adducts. Nevertheless, it is also important to note that clonal expansion of proliferating cells containing a *lacZ* mutation may augment MF, thereby increasing the apparent efficiency of total adducts. However, proportionally scaled increases in clonal expansion would be expected to equivalently augment the mutagenic efficiency of total adducts in all tissues. Indeed, recent next-generation sequencing of induced *lacZ* mutants failed to show cross-tissue variations in the proportion of the total mutants at a given dose resulting from clonal expansion (unpublished data).

The results presenteed in Figure 6 and Table 2 raise a range of interesting issues related to the *in vivo* toxicokinetics of 3-NBA, as well as tissue-specific difference in 3-NBA metabolism, processing of 3-NBA-induced damage, and tissue-specific replicative capacity. For example, the results show stark contrast between the effects of 3-NBA on lung tissue and FE1 cells, despite the fact that FE1 cells were derived from Muta™Mouse lung. More specifically, the mutagenic efficiency of total adducts is high for FE1 cells and zero for lung. This discrepancy is a direct consequence of the absence of *lacZ* mutants in lung tissue, and a significant increase in *lacZ* MF in FE1 cells (Figure 2 herein and Figure 2a and 4a in Arlt *et al.* (37)), despite the fact that total RAL was significantly elevated in both, and both are clearly exposed to an adduct that has been shown to be both persistent and promutagenic (i.e., dG-*N*^2^-3-ABA, Supplementary Table 1, available at *Mutagenesis* Online, (42,43,46)). Since FE1 cells retain numerous characteristics of alveolar epithelium (80), there is no reason to assert that the majority of the factors controlling the frequencies of mutations (e.g., capacity for repair, translesion synthesis, etc.) differ between Muta™Mouse lung tissue and FE1 cells. Thus, it seems reasonable to assert that the difference in adduct load is driven by cellular dose, and the difference in the mutagenic efficiency of total adducts is driven by the 100-fold difference in mitotic index (i.e., 14.1% versus 0.14%). With respect to the former, it is reasonable to emphasise that cells exposed *in vitro* will come into contact with a far greater amount of parent compound than an *in vivo* tissue that is remote from the site of first contact (i.e., GI tract). It is also necessary to acknowledge that the difference in adduct load at a given time point may also be driven by differences in exposures to reactive metabolites, and/or differences in damage processing. With respect to the former, Bendt-Weiss *et al.* (80) investigated changes in *cyp1a1*, *cy1a2* and *cyp1b1* gene expression in FE1 cells and lung tissue following exposures to benzo[*a*]pyrene (BaP). The results showed significant increases in *cyp1a1*, *cyp1a2* and *cyp1b1* expression in FE1 exposed *in vitro*, but no significant increases in either *cyp1a1* or *cyp1a2* in Muta™Mouse lung exposed *in vivo*. This difference, which may result from reduced *in vivo* exposure of metabolically-competent lung cells to a parent compound such as BaP, may also contribute to the reduced lung effect observed herein. Alternatively, Muta™Mouse lung may have a limited relative capacity to convert 3-NBA to the aforementioned metabolite *N*-hydroxy-3-aminobenzanthrone. This seems unlikely since the *in vivo* and *in vitro* analyses showed significant increases in 3-NBA adducts in Muta™Mouse lung tissue and cells derived from Muta™Mouse lung tissue.

The bootstrapped relationship presented in Figure 6 indicates that the mean of published lung MI values (i.e., 0.14 ± 0.044%) would correspond to a predicted mutagenic efficiency of total adducts of 0.068 ± 0.16, a value that is not significantly different from zero. Thus, as noted above, it seems highly likely that the inability of 3-NBA oral exposure to induce a significant increase in MF in the lung is primarily due to low MI. Shami and Evans (72) have indicated that although the proliferative indices of pulmonary cells varies according to cell type and animal age, the proportion of cells in adult rodents in M or S phase is generally very low. More specifically, MI values for adult rat bronchiolar/tracheal basal and nonciliated cells ranges from 0.05 to 0.25%. Similarly, the proportion of adult rat Clara and alveolar Type II cells in S phase ranges from 0.1 to 1.1% and 0.1 to 0.5%, respectively. Similar Clara cell values have been recorded for adult mouse and hamster (i.e., 0.16 and 0.3%, respectively). Nevertheless, some studies have recorded relatively high proportions of Type II cells in S phase (i.e., 1.1–2.9% in adult hamster). The earlier Arlt *et al.* (45) study that investigated 3-NBA-induced adducts and *lacZ* MF in Muta™Mouse following *ip* injection also did not detect significant increases in MF in the lung, in addition to kidney, spleen and testis. The authors of that study also highlight studies noting how the slow cell turnover in lung can reduce the likelihood of observing significant elevations in lung MF (73,74). Although the contrast between *in vivo* lung tissue results and FE1 cell results, presented both here and elsewhere, suggests that extrapolations from *in vitro* to *in vivo* should be done with caution, there is no evidence to support the notion that these differences are driven by important metabolic and/or genetic differences.

Although the adduct frequency in lung is relatively low, it is significantly above zero and comparable to that observed in the bone marrow, thus indicating that the lung is clearly exposed to reactive metabolites. Nevertheless, the low levels of 3-NBA-DNA adducts in lung, relative to liver, colon and bone marrow, suggest low exposure of Muta™Mouse lung to reactive 3-NBA metabolites. This result is supported by the Arlt *et al.* (45) study and similar results were obtained in a recent murine study of benzo[*a*]pyrene; DNA adduct levels in lung ~10-fold lower than liver 24 h after a single *i.p.* injection of 2 mg/kg body weight (70). However, this contrasts with the results of Long *et al*. (81) who noted statistically elevated levels of adducts in Muta™Mouse lung following 28-day repeat dose oral exposures to nine polycyclic aromatic hydrocarbons (PAHs), despite a lack of total adduct increases in bone marrow, liver, intestine, and/or glandular stomach for selected compounds (81). More specifically, for benzo[*a*]pyrene only, total adduct levels in lung were 1.8- to 3.3-fold greater than liver (81,82). The Arlt *et al.* findings also contrast rat studies that showed high levels of 3-NBA-induced adducts in lung following *i.p.* injection (45,75). The reason for these discrepancies across compounds, rodent species, and/or rodent strains, is not known; however, it seems likely that selected murine tissues lack the enzymatic capacity to carry out the initial nitroreduction that is essential for the formation of 3-NBA *N*-hydroxyarylamine intermediates, and ultimately the DNA-reactive metabolites harbouring nitrenium or carbenium ions (36,37). Nevertheless, this contention is not supported by Chen *et al.* (48), who confirmed that levels of 3-NBA reductase in Muta™Mouse lung are actually quite high relative to liver, colon, bone marrow and bladder.

It is also reasonable to hypothesise that lack of repair, and/or error-prone translesion synthesis (TLS), of 3-NBA-induced DNA damage could alter mutation fixation in tissues such as lung. Kucab *et al.* (44), recently suggested that the mutagenic potency of 3-NBA may be enhanced by an ability of 3-NBA adducts to avoid global genomic nucleotide excision repair. In addition, several studies have noted that DNA polymerase κ (kappa) can bypass BaP-induced dG lesions (i.e., BPDE-*N*^2^-dG) in a predominantly error-free manner (83–86). However, in contrast, Pande *et al.* (17) showed that Pol κ and Pol η are major contributors to mutagenic TLS of dG-C8-N-3-ABA adducts in human embryonic kidney 293T (HEK293T) cells. Interestingly, Ogi *et al.* (83) showed that both mouse and human *Polk* genes have upstream AhR (Ah-receptor) binding sites; possibly making gene expression and lesion bypass AhR-inducible. These authors further note that Pol κ can reduce the mutagenicity of some bulky PAH adducts in some tissues; with levels of POLk protein in lung enhanced following i.p. administration of 3-methlycholanthrene. This finding is supported by the work of Stancel *et al.* (87) who showed increased *lacI* MF in lung, liver and kidney of *Polk* knock-out BigBlue^®^ mice. Whether TLS in lung serves to augment or diminish the conversion of 3-NBA adducts to fixed mutations *in vivo* remains to be determined.

Total RAL levels in intestinal epithelium are also low, but they are significantly greater than zero, and this tissue was also clearly exposed. Given that published MI values for small intestine are often as high as bone marrow, the absence of a relationship between MF and total adducts was unexpected. Although published mitotic index values for intestinal epithelium vary according to intestinal segment and position within villi and crypts, values are generally in the 3–15% range. The geometric mean MI from four reviewed studies is 4.8 ± 1.5% (88–91), and based on the bootstrapped relationship presented in Figure 6, this value would correspond to a predicted mutagenic efficiency of total adducts of 0.52 ± 0.15, a value that would fall between colon and bone marrow (Figures 5 and 6). Using the RAL results presented in Figure 2, the predicted small intestine MF associated with 25, 50 and 75 mg/kg would be ~1.5 × 10^–5^, a value that would not be significantly greater than the concurrent controls (i.e., 9.4 ± 1.6 × 10^–5^ for 3 days and 18.2 ± 5.5 × 10^–5^ for 28 days). Taking into account the aforementioned factors that affect adduct formation and the mutagenic efficiency of total adducts, it seems reasonable to suppose that the absence of response in both lung and intestine are likely driven by a combination of low mitotic index (lung only), low levels of exposure to reactive metabolites, and tissue-specific differences in adduct processing. It should be noted that since the processing of small intestine tissue involved removal of luminal contents and tissue rinsing (see Methods and Materials), it seems unlikely that the results obtained (i.e., DNA adducts, but no mutations) are related to bacterial contamination of murine DNA samples.

Despite the aforementioned expectations, the difference between small intestine and other tissues observed herein (e.g., liver, colon and bone marrow) are consistent with earlier works that examined digestive tract effects of 3-NBA and other nitroarenes. The aforementioned Kohara *et al.* (77) study noted that intragastric administration of a dinitropyrene mixture readily induced large increases in *lacZ* MF in Muta™Mouse colon (Supplementary Table 2, available at *Mutagenesis* Online). Although small intestine was not examined in that study, the colon MF levels were more than 2-fold greater than those observed for stomach. Arlt *et al.* (45) suggest that the greater colonic effect of nitroarenes such as 3-NBA may be related to enhanced nitroreduction by colonic bacteria. They further note that the strong mutagenic effect of 3-NBA in Muta™Mouse colon following *i.p.* administration is consistent with reports of high adduct levels in the colon, relative to small intestine or stomach, in rats and mice exposed either orally or by i.p. injection (41,45,70,75).

The inability to observe significant increases in *lacZ* MF in the small intestine may also be related to a high frequency of induced apoptosis. It is well known that small intestine is exceptionally sensitive to radiation and chemical mutagens, displaying high rates of induced apoptosis in rapidly proliferating crypt cells (92–94); high frequency chemically induced apoptosis in intestinal epithelia may restrict the ability to recover induced *lacZ* mutants following oral exposures. Nevertheless, Lynch *et al.* (95) and Itoh *et al.* (96) did show significant increases in *lacZ* MF in Muta™Mouse small intestine following 4 or 5 daily oral exposures to PhIP, respectively (Supplementary Table 1, available at *Mutagenesis* Online). In addition, the aforementioned Long et al study of nine PAHs showed robust DNA damage and mutagenicity responses in Muta™Mouse small intestine following 28-day oral exposures (81). However, it should be emphasised that each of the highlighted studies examined intestinal tissues after several daily administrations; whereas, the current study examined tissues after a single acute dose. Interestingly, a study of urethane by Singer (97) detected significant increases in *lacZ* MF in small intestine only following consecutive daily treatments for 28 or 56 days; treatments for 7 days did not induce a significant MF increase.

### Conclusions and implications

The quantitative relationship between total DNA damage frequency and induced MF has rarely been explored. In an ideal situation, sufficient information on the quantitative relationship between adduct frequency (i.e., a marker of exposure and internal dose) and MF (i.e., an adverse effect on the exposed tissue), for noteworthy environmental mutagens, would permit use of the former to determine the risk of the latter; moreover, the risk of adverse health effects that have been mechanistically associated with the latter (i.e., cancer, heritable genetic disorders). As noted, MF will be a function of the production of DNA-reactive metabolites, DNA damage frequency, the probability of repair and the probability that the damaged site will be misread during replication. The latter factor implies that the frequency of chemically-induced mutations will be dependent on the proliferation index of damaged cells in the exposed tissue. The results presented here confirm that there is a significant positive effect of tissue-specific mitotic index on the maximum observed tissue-specific mutagenic efficiency of total adducts. However, some tissues, such as small intestine, do not follow the observed empirical relationship. In this tissue, the MF observed at selected sampling time(s) following an acute 3-NBA exposure does not appear to be empirically related to MI. In contrast, the mutagenic efficiency of total adducts for other tissues (i.e., bone marrow) is higher than that predicted by the empirical relationship shown in Figure 6. In the latter case, clonal expansion of cells containing a mutant *lacZ* may be increasing the measured MF and the apparent mutagenic efficiency of total adducts; nevertheless, clonal expansion of mutants is likely having a proportionally scaled effect on other tissues. It should be noted that the mutagenic efficiency of total adducts, particularly for slowly proliferating tissues, may also be influenced by cellular replication induced by the cytotoxicity of the tested compound. For example, Tombolan *et al*. (98) noted that the marked increases in hepatic MF of Muta™Mouse exposed to elevated levels 5,9-dimethydibenzo[*c,g*]carbazole (DMDBC) corresponded with increased levels of cytotoxicity-induced cellular proliferation.

The restricted nature of the data presented in Figure 6 diminishes the ability to make general statements regarding the interpretation of DNA adduct data in a risk assessment context. Although tissue-specific frequencies of DNA adducts do provide an indication of target tissue dose, the risk of adverse effect in tissues with relatively high proliferative capacity can be diminished in some tissues relative to other similar tissues (e.g., small intestine versus colon). The reasons underlying these differences are unclear and may be compound-specific, with variations in tissue-specific handling of DNA damage almost certainly altering the tissue-specific risk of mutation. Nevertheless, it is important to emphasise that an absence of tissue-specific, promutagenic DNA adducts will be associated with an absence of risk.

Although the results presented support the assertion that the risk of tissue-specific mutation formation is dependent, in part, on damage frequency and proliferative capacity, the likely roles of compound-specific distribution and metabolism, tissue-specific damage processing, exposure regime and post-exposure sampling time, intratissue variations in replicative capacity, and the nature and magnitude of tissue-specific responses to toxic insults, indicate that further work is required prior to any general statement regarding the ability to interpret adduct frequency in a disease risk context (e.g., additional tissues, additional cells *in vitro*, wider range of sampling times). In addition, the empirical analyses of the relationship between the maximum observed mutagenic efficiency of total adducts and MI (i.e., Figure 6; Table 2) indicate that more doses are required to obtain robust results for slowly proliferating tissues (e.g., liver). It seems likely that the nature and magnitude of empirical relationships between the mutagenic efficiency of total adducts and proliferative index will depend on compound type, making it possible to make general statements for distinct classes of environmental mutagens.

This work provides a proof of principle regarding the use of the Muta™Mouse system to critically examine the health risks associated with tissue-specific total adduct loads, which will be employed in future studies to provide a stronger context for the interpretation of DNA adduct frequency values for selected classes of environmental mutagens. Indeed, we are already scrutinising the relationships between induced MF and total adduct frequency for a series of PAHs.

## Supplementary data

Supplementary data are available at *Mutagenesis* Online.

## Funding

This work was funded by Health Canada intramural funding (P.A.W. and G.R.D.). Cancer Research UK (D.H.P. and V.M.A., grant C313/A14329) and Wellcome Trust (D.H.P. and V.M.A., grants 101126/Z/13/Z and 101126/B/13/Z). D.H.P. and V.M.A. are members of the Wellcome Trust funded COMSIG (Causes of Mutational SIGnatures) consortium.

## Supplementary Material

Supplementary Material
