# Supplementary material for: Quantitative relationships between lacZ mutant frequency and DNA adduct frequency in Muta™Mouse tissues and cultured cells exposed to 3-nitrobenzanthrone
Source: Mutagenesis. 2017 Jan 17;32(2):299–312. doi: 10.1093/mutage/gew067 (PMC5638019; doi:10.1093/mutage/gew067)
Supplement: Supplementary Material [file gew067_suppl_supplementary_material.docx]

**FIGURE LEGENDS**

**Figure S1 –**

The relative proportions of each 3-NBA DNA adduct in the autoradiographic profiles (i.e., spots 1-5). Adduct profiles are shown for *in vivo* tissues collected 18 hr after single oral doses, and for FE1 cells *in vitro* collected 72 hrs after 6 hr exposures. RAL- relative adduct labelling. Error bars show one standard error of the mean. The identities of the individual adducts are described in the text. Adduct proportions not shown indicate lack of detection. A-bone marrow, B-colon, C-small intestine, D-liver, E-lung, F-FE1 cells.

**Figure S1 – Panel A (bone marrow)**

**A**

**Figure S1 – Panel B (colon)**

**Figure S1 – Panel C (intestine)**

**Figure S1 – Panel D (liver)**

**Figure S1 – Panel E (lung)**

**Figure S1 – Panel F (FE1 cells)**

**Table S1.** The persistence of DNA adducts induced by intratracheal rat exposure to 3-NBA. Persistence values, expressed as half-life in days or weeks, were calculated using temporal adduct frequency data presented in the Supplementary Material of Bieler et al (2007) *Carcinogenesis* **28**:1117–1121. Spot 3 (dG-*N*^2^-ABA) was the most abundant and persistent adduct; the total includes the 5 adducts detected using ^32^P-postlabelling. The quality of the decay data for other adducts is highly variable. Consequently, in most instances, the half-life values could not be reliably calculated. Nevertheless, the available data indicate that the dG-*N*^2^-ABA adduct is 4- to 9-fold more persistent than the other adducts. Additional analyses confirmed that the persistence of the dG-*N*^2^-ABA adduct is significantly greater (p<0.01) than total adduct persistence. ND – not determined due to lack of a statistically significant trend in the decline of adduct abundance over the duration of the study (36 weeks).

| **Tissue** | **Half-life - Spot 3 (dy)** | **Half-Life - Total Adducts (dy)** |
| --- | --- | --- |
| Lung | ND | 126.5 |
| Pancreas | 169.9 | 83.4 |
| Kidney | ND | 168.2 |
| Bladder | 137.5 | 95.2 |
| Heart | 99.4 | 61.4 |
| Small Intestine | 160.4 | 100.8 |
| **Average ± SEM** | **141.8 ± 15.7 days** | **105.9 ± 15.2 days** |
|  | **20.3 ± 2.2 weeks** | **15.1 ± 2.2 weeks** |

**Table S2.** Summary of positive Muta™Mouse responses in tissues that yielded negative results for 3-NBA. The results presented, which were extracted from the scientific literature, are restricted to homo- or heterocyclic PACs (polycyclic aromatic compounds) that require metabolic conversion to a reactive electrophile. Maximum mutant frequency indicates fold change over concurrent control. Intestine – small intestine, ip – intraperitoneal injection. ig – intragastric injection.

| **Compound** | **Tissue** | **Administration Route** | **Administration Time (days)** | **Sampling Time (days)** | **Maximum Dose (mg/kg)** | **Maximum Mutant Frequency** | **Source** |
| --- | --- | --- | --- | --- | --- | --- | --- |
| Benzo[*a*]pyrene | Lung | Gavage | 5 | 182 | 125 | 7.5-fold | 1 |
| Benzo[*a*]pyrene | Lung | Gavage | 5 | 14 | 125 | 4.1-fold | 2 |
| Benzo[*a*]pyrene | Lung | Gavage | 5 | 14 | 125 | 16.5-fold | 3 |
| Benzo[*a*]pyrene | Lung | Gavage | 12 | 14 | 10.4 | 4.4-fold | 3 |
| Benzo[*a*]pyrene | Lung | Gavage | 5 | 56 | 125 | 6.1-fold | 4 |
| Benzo[*a*]pyrene | Lung | Gavage | 5 | 14 | 125 | 4.4-fold | 5 |
| Benzo[*a*]pyrene | Lung | Gavage | 28 | 3 | 75 | 16.4-fold | 6 |
| 1,10-diazachrysene | Lung | *ip* | 28 | 7 | 14.3 | 3.9-fold | 7 |
| 4,10-diazachrysene | Lung | *ip* | 28 | 7 | 28.6 | 17.0-fold | 7 |
| 7,12-dimethylbenz[*a*]anthracene | Lung | *ip* | 28 | 0 | 0.7 | 4.0-fold | 8 |
| Benzo[*h*]quinoline | Lung | *ip* | 4 | 14 | 100 | 1.8-fold | 9 |
| Chrysene | Lung | *ip* | 28 | 7 | 28.6 | 2.8-fold | 7 |
| Benz[*a*]anthracene | Lung | Gavage | 28 | 3 | 80 | 1.6-fold | 6 |
| Dibenz[*a,h*]anthracene | Lung | Gavage | 28 | 3 | 25 | 28.1-fold | 6 |
| Benzo[*b*]fluoranthene | Lung | Gavage | 28 | 3 | 100 | 4.7-fold | 6 |
| Benzo[*k*]fluoranthene | Lung | Gavage | 28 | 3 | 100 | 2.4-fold | 6 |
| Indeno[1,2,3-cd]pyrene | Lung | Gavage | 28 | 3 | 50 | 1.7-fold | 6 |
| Benzo[*ghi*]perylene | Lung | Gavage | 28 | 3 | 25 | 2.0-fold | 6 |
| Dibenzo[*a,l*]pyrene | Lung | Gavage | 28 | 3 | 2 | 6.8-fold | 6 |
| Dinitropyrenes^*^ | Lung | *ig* | 28 | 7 | 57 | 1.6-fold | 10 |
| PhIP | Intestine | Gavage | 4 | 7 | 20 | 4.2-fold | 11 |
| PhIP | Intestine | Gavage | 5 | 14 | 20 | 5.0-fold | 12 |
| Benzo[*a*]pyrene | Intestine | Gavage | 5 | 14 | 125 | 24.2-fold | 2 |
| Benzo[*a*]pyrene | Intestine | Gavage | 1 | 14 | 100 | 4.8-fold | 13 |
| Benzo[*a*]pyrene | Intestine | Diet | 21 | 14 | 4.8 | 2.5-fold | 14 |
| Benzo[*a*]pyrene | Intestine | Diet | 28 | 14 | 4.8 | 3.2-fold | 14 |
| Benzo[*a*]pyrene | Intestine | Diet | 56 | 14 | 4.8 | 3.4-fold | 14 |
| Benzo[*a*]pyrene | Intestine | Gavage | 28 | 3 | 75 | 67.3-fold | 6 |
| Chrysene | Intestine | Gavage | 28 | 3 | 100 | 1.8-fold | 6 |
| Benz[*a*]anthracene | Intestine | Gavage | 28 | 3 | 80 | 1.7-fold | 6 |
| Dibenz[*a,h*]anthracene | Intestine | Gavage | 28 | 3 | 25 | 169.7-fold | 6 |
| Benzo[*b*]fluoranthene | Intestine | Gavage | 28 | 3 | 100 | 139.0-fold | 6 |
| Benzo[*k*]fluoranthene | Intestine | Gavage | 28 | 3 | 100 | 5.5-fold | 6 |
| Indeno[1,2,3-cd]pyrene | Intestine | Gavage | 28 | 3 | 50 | 26.4-fold | 6 |
| Benzo[*ghi*]perylene | Intestine | Gavage | 28 | 3 | 25 | 1.9-fold | 6 |
| Dibenzo[*a,l*]pyrene | Intestine | Gavage | 28 | 3 | 2 | 7.6-fold | 6 |

^*^20.2% 1,3-dinitropyrene, 30.4% 1,6-dinitropyrene, 35.2% 1,8-dinitropyrene, 14.2% unidentified isomer(s)

1. Hakura A, Tsutsui Y, Sonoda J, Mikami T, Tsukidate K, Sagami F, Kerns WD. 1999. Multiple organ mutation in the lacZ transgenic mouse (Muta mouse) 6 months after oral treatment (5 days) with benzo[a]pyrene. Mutat Res. 426:71-77.
2. Hakura A, Tsutsui Y, Sonoda J, Kai J, Imade T, Shimada M, Sugihara Y, Mikami T. 1998. Comparison between in vivo mutagenicity and carcinogenicity in multiple organs by benzo[a]pyrene in the lacZ transgenic mouse (Muta™Mouse). Mutat Res. 398:123-130.
3. Kosinska W, von Pressentin MD, Guttenplan JB. 1999. Mutagenesis induced by benzo[a]pyrene in lacZ mouse mammary and oral tissues: comparisons with mutagenesis in other organs and relationships to previous carcinogenicity assays. Carcinogenesis 20:1103-1106.
4. Guttenplan JB, Chen M, Kosinska W, Thompson S, Zhao Z, Cohen LA. 2001. Effects of a lycopene-rich diet on spontaneous and benzo[a]pyrene- induced mutagenesis in prostate, colon and lungs of the lacZ mouse. Cancer Lett 164:1-6.
5. Yamada K, Suzuki T, Kohara A, Hayashi M, Hakura A, Mizutani T, Saeki K. 2002. Effect of 10-aza-substitution on benzo[a]pyrene mutagenicity in vivo and in vitro. Mutat Res. 521:187-200.
6. Long AS, Lemieux, CL, Arlt, VM, White, PA. 2016. Tissue-specific in vivo genetic toxicity of nine polycyclic aromatic hydrocarbons assessed using the Muta™Mouse transgenic rodent assay. Toxicol Appl Pharmacol. 290:31-42.
7. Yamada, K, Suzuki, T, Kohara, A, Kato, TA, Hayashi, M, Mizutani, T, Saeki, K. 2004. In vivo mutagenicity of benzo[f]quinoline, benzo[h]quinoline, and 1,7-phenanthroline using the lacZ transgenic mice. Mutat Res. 559:83-95.
8. Hashimoto, K, Ohsawa, K, Kimura, M. 2004. Mutations induced by 4-(methylnitrosamino)-1-(3-pyridyl)-1-butanone (NNK) in the lacZ and cII genes of Muta™Mouse. Mutat Res. 560:119-131.
9. Yamada, K, Suzuki, T, Kohara, A, Hayashi, M, Mizutani, T, Saeki, K. 2004. In vivo mutagenicity of benzo[f]quinoline, benzo[h]quinoline, and 1,7-phenanthroline using the lacZ transgenic mice. Mutat Res. 559:83-95.
10. Kohara A, Suzuki T, Honma M, Oomori T, Ohwada T, Hayashi M. 2002. Dinitropyrenes induce gene mutations in multiple organs of the lambda/lacZ transgenic mouse (Muta™Mouse). Mutat Res. 515:73-83.
11. Lynch AM, Gooderham NJ, Boobis AR. 1996. Organ distinctive mutagenicity in MutaMouse after short-term exposure to PhIP. Mutagenesis 11:505-509.
12. Itoh, T, Kuwahara, T, Suzuki, T, Hayashi, M, Ohnishi, Y. 2003. Regional mutagenicity of heterocyclic amines in the intestine: mutation analysis of the cII gene in lambda/lacZ transgenic mice. Mutat Res 539:99-108.
13. Cosentino L, Heddle JA. 1999. A comparison of the effects of diverse mutagens at the lacZ transgene and Dlb-1 locus in vivo. Mutagenesis 14:131-119.
14. Cosentino L, Heddle JA. 2000. Differential mutation of transgenic and endogenous loci in vivo. Mutat Res. 454:1-10.
